# Treatment Patterns, Outcome, and Quality of Life of Patients With Extensive-Stage SCLC Receiving Third-Line Therapy—Data From the German CRISP Registry (AIO-TRK-0315): A Brief Report

**DOI:** 10.1016/j.jtocrr.2026.100959

**Published:** 2026-01-24

**Authors:** Maximilian Rost, Rieke Fischer, Martin Reck, Thomas Gauler, Cornelius Waller, Petros Christopoulos, Petra Hoffknecht, Martina Jänicke, Annette Fleitz, Andrea Härzschel, Carolin Lennartz, Paula Ludwig, Annika Groth, Michael Bendel, Jan Stratmann, Frank Griesinger, Michael Thomas, Wilfried E.E. Eberhardt, Martin Sebastian

**Affiliations:** a Department of Medicine II, Hematology and Oncology, Goethe University Frankfurt, University Hospital, Frankfurt, Germany; b Department I of Internal Medicine, Lung Cancer Group Cologne, University Hospital Cologne, Cologne, Germany; c Department of Thoracic Oncology, LungenClinic Grosshansdorf, Airway Research Center North (ARCN), German Center for Lung Research (DZL), Giessen, Germany; d Department of Radiation Oncology, University Hospital Essen, Essen, Germany; e Department of Medical Oncology, University Medical Center Freiburg, Faculty of Medicine, University of Freiburg, Freiburg, Germany; f Department of Thoracic Oncology, Thoraxklinik, University Hospital Heidelberg and Translational, Lung Research Center Heidelberg (TLRC-H), Member of the German Center for Lung Research (DZL), Heidelberg, Germany; g Department of Hematology/Oncology, Niels-Stensen-Kliniken Franziskus Hospital Harderberg, Hardenberg, Germany; h Clinical Epidemiology and Health Economics, iOMEDICO, Freiburg, Germany; i Statistics, iOMEDICO, Freiburg, Germany; j AIO-Studien-gGmbH, Berlin, Germany; k MVZ West, Würselen, Germany; l Oncology, University Hospital Saarland, Homburg, Germany; m Department of Hematology and Oncology, University Department Internal Medicine-Oncology, Pius-Hospital, University Medicine Oldenburg, Oldenburg, Germany; n Department of Medical Oncology, West German Cancer Center, University Hospital Essen, Essen, Germany; o Frankfurt Cancer Institute, Goethe University, Frankfurt am Main, Germany

**Keywords:** SCLC, Real-world data, Outcome research, Quality of life

## Abstract

**Introduction:**

Recurrent extensive-stage SCLC (ES-SCLC) remains a major therapeutic challenge. Although immune checkpoint inhibitors (2Ls), bispecific antibodies, and antibody-drug conjugates offer promise in early lines, outcomes beyond the second line (2L) remain poor. Real-world data on third line (3L) treatment patterns, clinical outcomes, and quality of life (QoL) after ICI-based first line (1L) therapy are limited but critical to inform clinical decision-making.

**Methods:**

CRISP is a prospective, multicenter registry for patients with lung cancer in Germany. In-depth patient and tumor characteristics, details about treatments, outcome, and patient-reported outcomes data are collected. Between September 2019 and April 2021, 114 sites in Germany recruited 518 patients with ES-SCLC, of which 82 started 3L therapy.

**Results:**

Of 82 patients, 58.5% were male and 79.3% were below 70 years old. At 3L start, Eastern Cooperative Oncology Group performance score was 0 in 18.3%, 1 in 39.0%, and more than or equal to 2 in 19.5% (23.2% unknown). In the 1L setting, 68 patients (82.9%) received platinum-based chemotherapy plus ICI and 68 (82.9%) were platinum refractory (time to next treatment < 3 mo). Furthermore, 2L therapy consisted of topotecan (47.6%) and other chemotherapies (52.4%). The three most common 3L regimens were paclitaxel (22%), topotecan (22%), and cyclophosphamide, doxorubicin, vincristine (17.1%). Local radiotherapy was used in 19 patients (23.2%) in the 3L. Median real-world progression-free survival was 2.2 months (95% confidence interval: [1.8–3.0]), and real-world overall survival was 4.4 months (95% confidence interval: [3.4–5.2]). Exploratory analyses revealed numerically longer real-world overall survival with increasing time between 1L and 2L initiation (<6 mo: 3.4 mo; 6–11 mo: 4.4 mo; ≥ 12 mo: 8.3 mo) and in patients with Eastern Cooperative Oncology Group performance score 0 to 1 versus more than or equal to 2 (5.1 versus 4.4 mo). QoL, assessed by FACT-L subscales, remained stable during follow-up.

**Conclusions:**

Outcomes in 3L therapy in ES-SCLC remain poor among subgroups, even in predominantly ICI-pretreated patients, strongly highlighting the need for better 3L strategies. Although QoL can be preserved, therapeutic efficacy is lacking. These real-world data are critical to support shared decision-making, helping clinicians and patients weigh the limited benefits of further chemotherapy against best supportive care.

## Introduction

Lung cancer is the most frequently diagnosed malignancy worldwide with SCLC accounting for approximately 15% of cases.^1^^,^^2^ SCLC is a high-grade neuroendocrine carcinoma characterized by rapid proliferation, early systemic spread, and poor prognosis.^3^ First-line (1L) treatment for extensive-stage disease (ES-SCLC) with platinum-based chemotherapy combined with immune checkpoint inhibitors (ICIs) achieves high initial response rates of approximately 60% to 70%.^3^ Recently, the bispecific T-cell engager tarlatamab demonstrated a significant overall survival (OS) benefit and will become standard of care (SoC) in second-line (2L) treatment.^4^^,^^5^ However, rapid widespread use may be constrained by organizational challenges, including cost and the need for inpatient management. Thus, cytotoxic chemotherapy remains a central treatment pillar beyond 1L.

Despite initial chemosensitivity, most patients experience rapid disease progression and resistance in later treatment lines.^3^ Therapeutic options beyond 2L remain limited, with no established SoC for third-line (3L) therapy. Real-world evidence to guide treatment decisions in this setting is sparse. This study contributes to that evidence by analyzing treatment patterns, outcomes, and quality of life (QoL) among patients with SCLC receiving 3L therapy, based on a large, prospective German-based registry.

## Materials and Methods

CRISP (NCT02622581) is a prospective, open, noninterventional, multicenter lung cancer registry collecting detailed patient and tumor characteristics, treatment data, outcomes, and patient-reported outcomes (PROs) from more than 190 cancer sites across Germany. Simultaneous participation in other clinical trials is permitted. Further methodological details have been published previously.^6^

For this analysis, eligible patients were aged above or equal to 18 years with histologically confirmed ES-SCLC who progressed during 2L therapy and initiated 3L treatment. Written informed consent was obtained from all patients.

Descriptive statistics summarized patient and treatment characteristics. OS was defined as the time from 3L initiation to death from any cause; patients alive or lost to follow-up at data cutoff (February 28, 2024) were censored at last contact. Real-world progression-free survival (rwPFS) was defined as time from 3L initiation to documented progression or death, with censoring at start of next line or last follow-up. OS and rwPFS were analyzed using Kaplan-Meier estimates. All analyses were performed using SAS (version [v]9.4).

PROs were assessed with the 36-item Functional Assessment of Cancer Therapy—Lung (FACT-L, v4), including the 27-item FACT-G core and a nine-item lung cancer subscale. The FACT-L has a possible total score range of 0 to 136. Questionnaires were administered at baseline (inclusion into the registry at 1L), every 2 months until month 12, and then every 3 months up to 36 months. PRO assessment time points are fixed and not linked to the patient’s current treatment line; thus, information on treatment at the time of PRO completion is not available. For this analysis, responses to month 15 were included and analyzed per scoring manual.

## Results

Between September 2019 and April 2021, 114 sites in Germany recruited 518 patients with ES-SCLC, of which 82 started 3L therapy and are included in this study (Supplementary Fig. 1). At 3L initiation, the median age was 64.4 years; 20.7% were older than 70 years and 58.5% were male (Table 1). The most common Eastern Cooperative Oncology Group (ECOG) performance score at 3L was 1 (39.0%). Comorbidities were present in 75.6%, with a Charlson Comorbidity Index (CCI) of 0 to 1 in 89.0% and more than or equal to 2 in 7.3%. At 3L initiation, most had brain (54.9%), bone (54.9%), and liver (57.3%) metastases.

**Table 1:** Demographic and Clinical Characteristics and Treatment Patterns Before 3L

Patient Characteristics	n (%), N = 82
Age at 3L initiation, median (25%–75% quartile)	64.4 (60.0–69.1)
Sex	
Female	34 (41.5)
Male	48 (58.8)
Smoking status at inclusion	
History of smoking	76 (92.7)
No history of smoking	3 (3.7)
Missing	3 (3.7)
ECOG at 3L initiation	
0	15 (18.3)
1	32 (39)
≥ 2	16 (19.5)
Missing	19 (23.2)
BMI at inclusion (kg/m^2^), mean (±SD)	26.1 (5.63)
CCI (0–24) at inclusion	
0	53 (64.6)
1	23 (28)
2	2 (2.4)
3	4 (4.9)
Site of metastasis at 3L initiation	
Brain	45 (54.9)
Bone	45 (54.9)
Liver	47 (57.3)
Lung (contralateral)	17 (20.7)
Distant lymph node	42 (51.2)
Adrenal gland	31 (37.8)
Pleura	24 (29.3)
Treatment Characteristics Before 3L	
1L treatment	
Carboplatin, etoposide, atezolizumab	61 (74.4)
Carboplatin, etoposide	10 (12.2)
Cisplatin/carboplatin, etoposide, atezolizumab	4 (4.9)
Cisplatin/carboplatin, etoposide	2 (2.4)
Cisplatin, etoposide	2 (2.4)
Cisplatin, etoposide, atezolizumab	1 (1.2)
Carboplatin, etoposide, paclitaxel, atezolizumab	1 (1.2)
Carboplatin, etoposide, vincristine, atezolizumab	1 (1.2)
2L treatment	
Topotecan	39 (47.6)
Cyclophosphamide, doxorubicin, vincristine	11 (13.4)
Carboplatin, etoposide	9 (11)
Carboplatin, etoposide, atezolizumab	6 (7.3)
Carboplatin, paclitaxel	4 (4.9)
Cyclophosphamide, epirubicine, vincristine	4 (4.9)
Atezolizumab	3 (3.7)
Carboplatin, bendamustin	3 (3.7)
Carboplatin, etoposide, lurbinectidin	3 (3.7)
Radiotherapy before 3L	
Yes	60 (73.2)
Conventional fractionated radiotherapy	57 (45.6)
Stereotactic ablative radiotherapy	19 (15.2)
Chemoradiotherapy	10 (8)
Brachytherapy	12 (9.6)
Other	14 (11.2)
Missing	19 (15.2)
No	21 (25.6)
1L platinum sensitivity status	
CFI < 90 d	68 (82.9)
CFI ≥ 90 to < 180 d	11 (13.4)
Time from start 1L to start 3L	
<6 mo	9 (11)
6–11 mo	36 (43.9)
12–24 mo	32 (39.1)
>24 mo	5 (6.1)

1L, first line; 2L, second line; 3L, third line; BMI, body mass index; CCI, Charlson Comorbidity Index; CFI, chemotherapy-free interval; ECOG, Eastern Cooperative Oncology Group.

In the 1L setting, 82.9% received platinum-based chemotherapy combined with ICI (Table 1). The most common 2L therapies were topotecan in 39 patients (47.6%) and cyclophosphamide/doxorubicin/vincristine in 10 patients (12.2%). Surgery and radiotherapy in limited stage or as local treatment modalities before 3L initiation were used in six (7.3%) and 60 (73.2%) patients, respectively. Platinum-resistant disease—defined as chemotherapy-free interval less than 90 days to start of 2L—was present in 68 patients (82.9%), and most (54.9%) received 3L therapy less than a year after start of 1L.

In the 3L setting, 18 patients (22%) received either paclitaxel or topotecan, whereas cyclophosphamide/doxorubicin/vincristine was used in 14 patients (17.1%) (Table 2). The sequences of treatment strategies from 1L to 3L are found in Supplementary Table 1. Radiotherapy was used in 19 patients (23.2%), whereas surgery was used in none.

**Table 2:** Treatment Patterns and Effectiveness in 3L

Treatment Characteristics	n (%), N = 82
Paclitaxel	18 (22)
Topotecan	18 (22)
Cyclophosphamide, doxorubicin, vincristine	14 (17.1)
Carboplatin, etoposide	8 (9.8)
Ipilimumab, nivolumab	5 (6.1)
Carboplatin, paclitaxel	4 (4.9)
Atezolizumab	2 (2.4)
Cyclophosphamide, epirubicin, vincristine	2 (2.4)
Docetaxel	2 (2.4)
Gemcitabine, vinblastin	2 (2.4)
Other	3 (3.7)
Radiotherapy in 3L	
Yes	19 (23.2)
No	62 (75.6)
Missing	1 (1.2%)

**Treatment Effectiveness**	
Best response to 3L treatment	
CR	0 (0)
PR	7 (8.5)
SD	13 (15.9)
PD	29 (35.4)
Missing	30 (36.5)
3L not yet completed	3 (3.7)

3L, third line; CR, complete response; 3L, third line; PD, progressive disease; PR, partial response.

In real-world assessments, no complete responses were found, partial response occurred in 8.5%, stable disease in 15.9%, and progression in 35.4%, with response undocumented in 36.5%.

**Figure 1:**
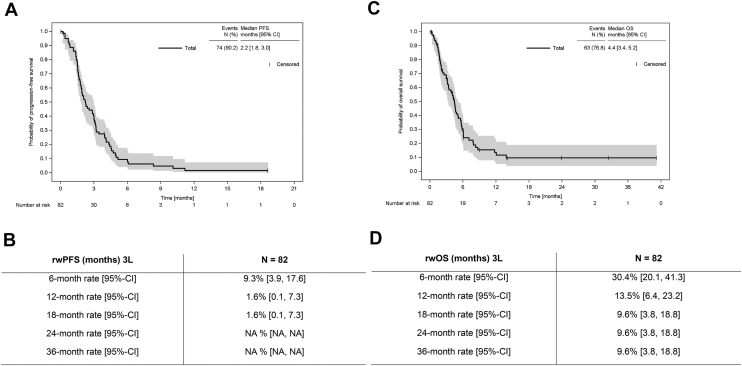
Kaplan-Meier curve for rwPFS in 3L therapy (*A*), landmark rwPFS rates for patients receiving 3L therapy (*B*), Kaplan-Meier curve for rwOS in 3L therapy (*C*), landmark rwOS rates for patients receiving 3L therapy (*D*). 3L, third line; CI, confidence interval; NA, not available; rwOS, real-world overall survival; rwPFS, real-world progression-free survival.

Median rwPFS was 2.2 months (95% confidence interval [CI]: [1.8–3.0]) and rwOS 4.4 months (95% CI: [3.4–5.2]) (Fig. 1*A*–*D*). Exploratory subgroup analyses revealed longer rwOS in patients with a longer interval between 1L and 2L initiation; 3.4 months for less than 6 months, 4.4 months for 6 to 11 months, and 8.3 months for more than or equal to 12 months. Patients with ECOG 0 to 1 had numerically longer rwOS compared with ECOG more than or equal to 2 (5.1 versus 4.4 mo) (Supplementary Figs. 2 and 3).

PRO data were documented for 63 patients (75.6%). Mean changes from baseline in physical, social/family, emotional, and functional well-being, including the Lung Cancer Subscale, were small and did not reach clinically meaningful thresholds in the course of 3L therapy (Supplementary Fig. 4).

## Discussion

In this study, we report on treatment patterns, clinical outcomes, and QoL in a contemporary real-world cohort of 82 German patients with ES-SCLC treated with 3L systemic therapy. Notably, the vast majority (82.9%) had previously received ICI as part of 1L chemo-immunotherapy, reflecting the rapid uptake of this new standard after its approval in March 2019.

Thus, our cohort represents a clinically relevant population treated with current SoC regimens. As the only approved 2L option in the European Union, topotecan was the most common treatment for progressing patients.

However, treatment regimens in the 3L setting were heterogeneous, with paclitaxel, topotecan, and cyclophosphamide/doxorubicin/vincristine each used in approximately one-quarter of patients. A recently published real-world study from the United States revealed a higher rate of ICI treatment and lurbinectedin in 3L, a drug that is not currently approved in the European Union.^7^ In the pre-ICI era, taxane-based chemotherapy and platinum rechallenge were the most frequently used regimens, with more than 10 different therapies reported across cohorts, underscoring the lack of a standardized approach in later-line treatment of SCLC.^8^

The observed median rwOS of 4.4 months is consistent with retrospective data from both ICI-era and pre-ICI real-world cohorts. For example, an equally sized recent U.S. cohort of ICI-pretreated patients with SCLC reported a median rwOS of 4.3 months after 3L therapy.^7^ Historical data before the ICI era similarly reported median rwOS between 4.4 and 5.8 months.^8^^,^^9^

Although outcome was poor in all comparable studies, the heterogeneity of treatments among all cohorts makes it difficult to determine an optimal treatment sequence. Notably, best supportive care in the 3L is associated with a median OS of less than 1 month, but identifying patients who may benefit from 3L therapy remains challenging.^8^ In our subgroup analyses, initiation of 2L therapy within 6 months of 1L, suggestive of chemotherapy-resistant disease, was associated with shorter rwOS (3.4 mo), whereas patients with longer 1L to 2L intervals achieved a more meaningful benefit with a median rwOS of up to 8.3 months in 3L, consistent with more favorable tumor biology. Differences by ECOG performance score were modest, with rwOS differing in weeks, underscoring the importance of shared decision-making when considering 3L therapy.

Inclusion of QoL data in shared decision-making can help inform patients about systemic treatment options versus best supportive care. Prospectively collected PROs using the FACT-L questionnaire provide insights into the lived experience of patients with SCLC undergoing multiple lines of therapy. Baseline values were comparable to those reported in patients with advanced NSCLC.^6^^,^^10^ Subscales remained relatively stable, indicating acceptable treatment tolerability but little symptom improvement. Although assessments were collected at predefined intervals without line-specific annotation, these data nonetheless provide rare and valuable insights into PROs during later lines of SCLC therapy.

In the present cohort, a substantial proportion of patients (82.9%) receiving 3L therapy had platinum-resistant disease, reflecting the chemotherapy-refractory nature of SCLC and further limiting available treatment options. In this context, tarlatamab may offer an especially promising therapeutic alternative. In the DeLLphi-301 trial, tarlatamab demonstrated a response rate of 52% among platinum-resistant patients.^4^ As bispecific T-cell engagers advance into earlier treatment lines and may ultimately reshape the therapeutic paradigm, it remains uncertain how their unique mechanism will influence subsequent chemotherapy efficacy. In parallel, antibody-drug conjugates such as ifinatamab-deruxtecan have demonstrated encouraging activity in the 2L setting.^11^ Together, these developments underscore the ongoing efforts to translate molecular insights into more effective, mechanism-driven therapies. Our data set thus provides an important real-world benchmark for future patient populations treated in this evolving therapeutic landscape.

Our study has several limitations. The relatively small sample size limits the generalizability of our findings. The heterogeneity of systemic therapies, radiotherapy use, and prior treatments complicates outcome interpretation; however, they are indicative of real-world data.

Furthermore, missing effectiveness assessments in 36.5% of cases may have influenced response rate estimates. Despite these limitations, this prospective registry-based analysis provides valuable real-world insights into a population with urgent unmet medical need. Our data demonstrate the only moderate and not sustainable effectiveness of 3L treatment strategies in the ICI era. It remains an urgent need to introduce new treatment modalities in this hard-to-treat patient population.

## CRediT Authorship Contribution Statement

**Maximilian Rost:** Conceptualization & design, Writing - original draft, Writing - review & editing.

**Rieke Fischer:** Investigation, Resources, Writing - review and editing.

**Martin Reck:** Investigation, Resources, Writing - review and editing.

**Thomas Gauler:** Investigation, Resources, Writing - review and editing.

**Cornelius Waller:** Investigation, Resources, Writing - review and editing.

**Petros Christopoulos:** Investigation, Resources, Writing - review and editing.

**Petra Hoffknecht:** Investigation, Resources, Writing - review and editing.

**Martina Jänicke:** Conceptualization, Formal analysis, Methodology, Supervision, Visualization, Writing - original draft.

**Annette Fleitz:** Conceptualization, Data curation, Formal analysis, Methodology, Project administration, Visualization, Writing - original draft.

**Andrea Härtschel:** Conceptualization, Data curation, Formal analysis, Methodology, Project administration, Visualization, Writing - original draft.

**Carolin Lennartz:** Conceptualization, Data curation, Formal analysis, Methodology, Project administration, Visualization, Writing - original draft.

**Paula Ludwig:** Project administration, Writing - review and editing.

**Annika Groth:** Project administration, Writing - review and editing.

**Michael Bendel:** Investigation, Resources, Writing - review and editing.

**Jan Stratmann:** Conceptualization and design, Resources, Supervision, Writing - review & editing.

**Frank Griesinger:** Conceptualization, Funding acquisition, Investigation, Resources, Supervision, Writing - original draft.

**Michael Thomas:** Conceptualization, Funding acquisition, Investigation, Resources, Supervision, Writing - original draft.

**Wilfried E. E. Eberhardt:** Conceptualization, Funding acquisition, Investigation, Resources, Supervision, Writing - original draft.

**Martin Sebastian:** Conceptualization, Funding acquisition, Investigation, Resources, Supervision, Writing - original draft.
