# Supplementary material for: Treatment Patterns, Outcome, and Quality of Life of Patients With Extensive-Stage SCLC Receiving Third-Line Therapy—Data From the German CRISP Registry (AIO-TRK-0315): A Brief Report
Source: JTO Clin Res Rep. 2026 Jan 24;7(4):100959. doi: 10.1016/j.jtocrr.2026.100959 (PMC13066948; doi:10.1016/j.jtocrr.2026.100959)
Supplement: Supplementary Figure 1 [file mmc1.pptx]

## Slide 1
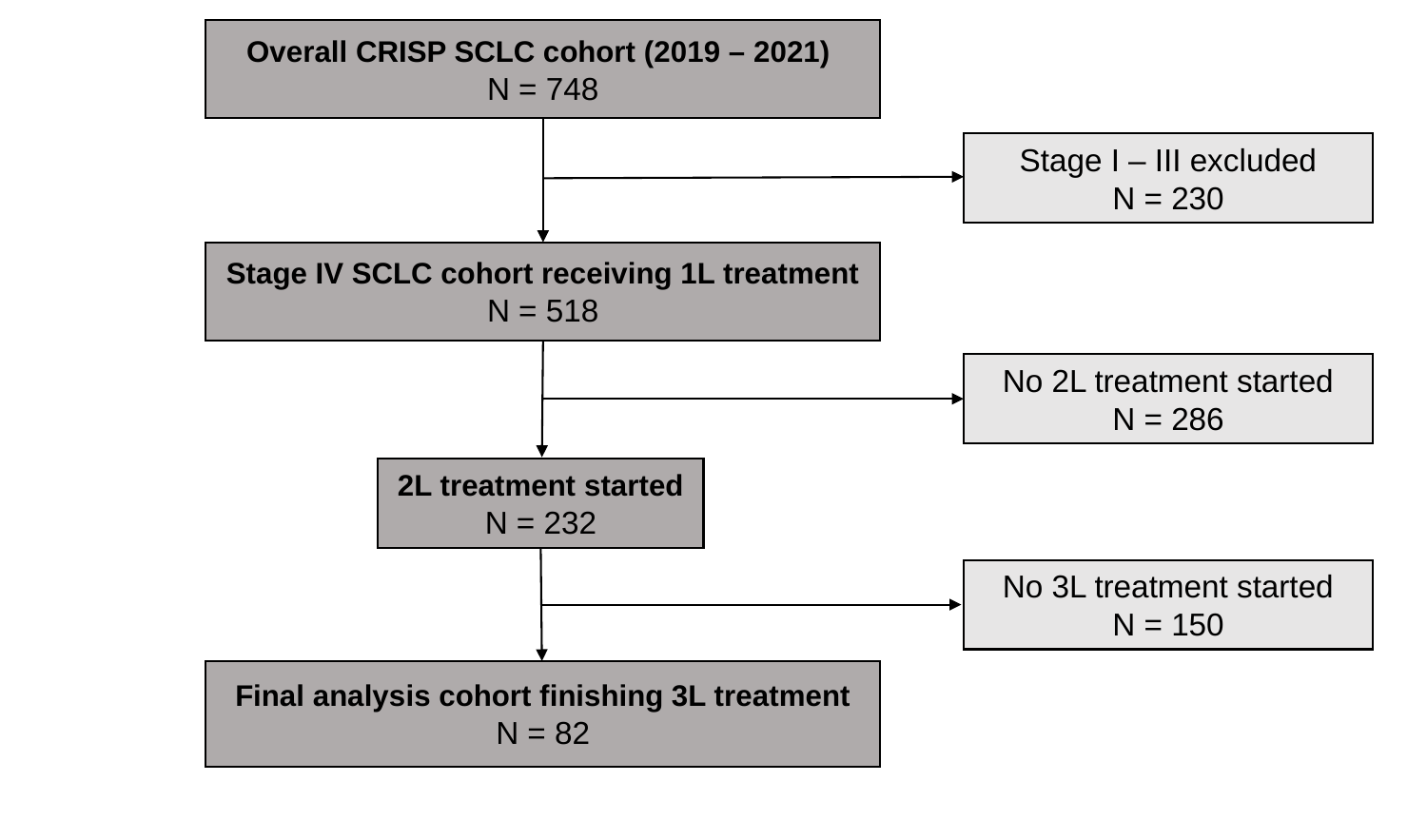

Overall CRISP SCLC cohort (2019 – 2021)
N = 748
Stage I – III excluded
N = 230
Stage IV SCLC cohort receiving 1L treatment
N = 518
No 2L treatment started
N = 286
2L treatment started
N = 232
No 3L treatment started
N = 150
Final analysis cohort finishing 3L treatment
N = 82
