# Supplementary figures and images for: Treatment Patterns, Outcome, and Quality of Life of Patients With Extensive-Stage SCLC Receiving Third-Line Therapy—Data From the German CRISP Registry (AIO-TRK-0315): A Brief Report
Source: JTO Clin Res Rep. 2026 Jan 24;7(4):100959. doi: 10.1016/j.jtocrr.2026.100959 (PMC13066948; doi:10.1016/j.jtocrr.2026.100959)

## Slide 1
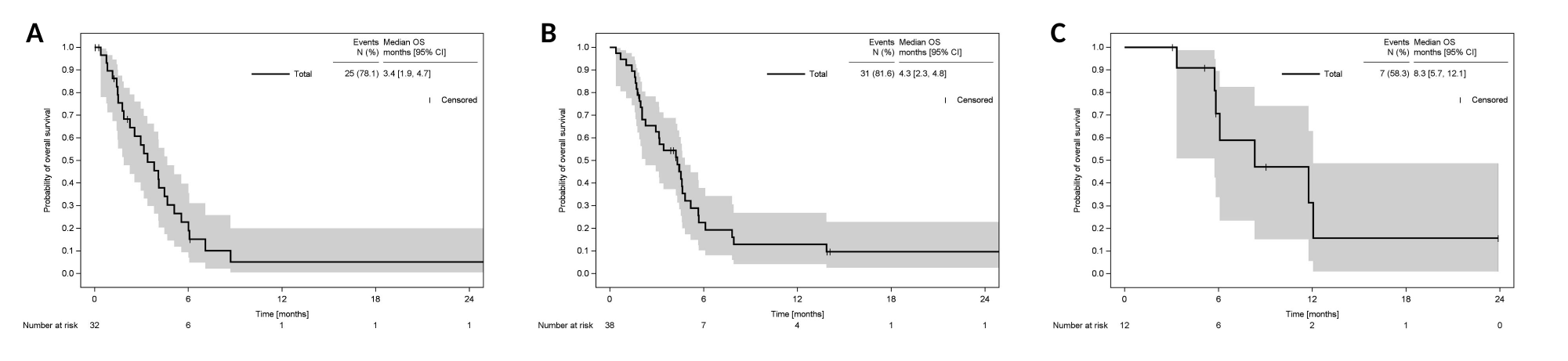

A
B
C

Supplement: Supplementary Figure 2 [file mmc2.pptx]

## Slide 1
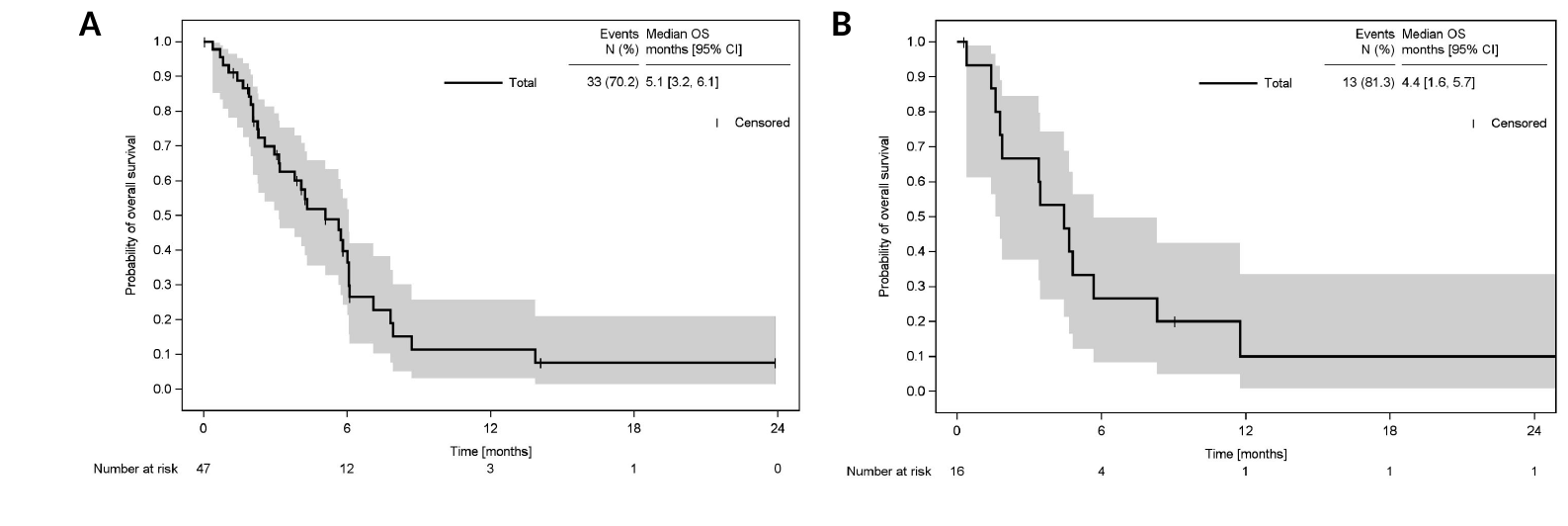

A
B

Supplement: Supplementary Figure 3 [file mmc3.pptx]

## Slide 1
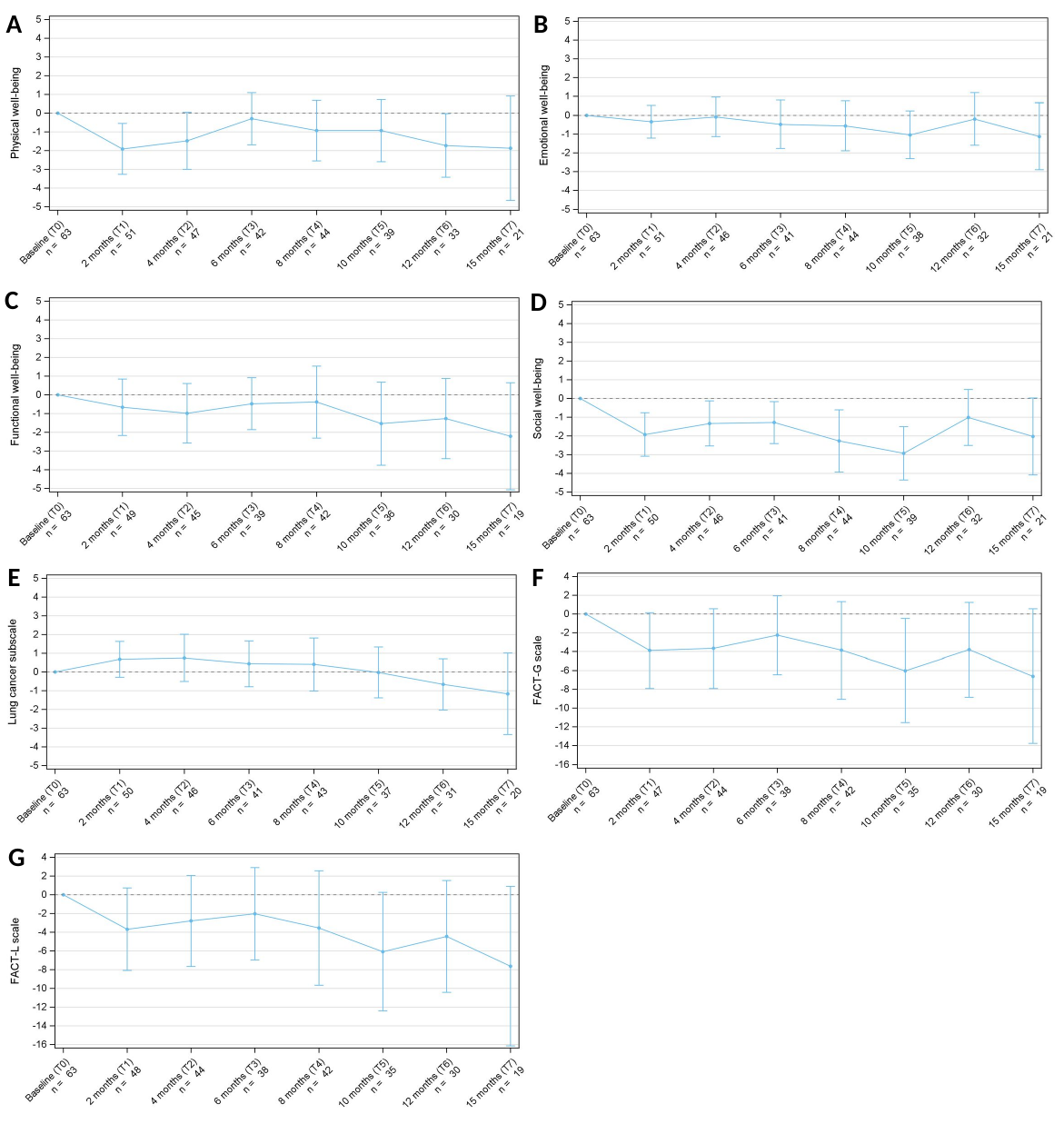

A
B
C
D
E
F
G

Supplement: Supplementary Figure 4 [file mmc4.pptx]
